# *In vitro* analysis of the pH stability of dental bleaching gels during in-office procedures

**DOI:** 10.4317/jced.57367

**Published:** 2021-01-01

**Authors:** Rafael-Pinto de Mendonça, Joberth-Rainner Baliza, Adrieli Burey, Larissa-Maria-Assad Cavalcante, Alessandro-Dourado Loguercio, Fernanda-Signorelli Calazans, Marcos-de Oliveira Barceleiro

**Affiliations:** 1 Fluminense Federal University (UFF), Nova Friburgo Health Institute, Operative Dentistry Department, Nova Friburgo, RJ, Brazil; 2 Ponta Grossa State University (UEPG), Ponta Grossa School of Dentistry, Operative Dentistry Department, Ponta Grossa, PR, Brazil; 3 Fluminense Federal University (UFF), School of Dentistry, Operative Dentistry Department, Niterói, RJ, Brazil

## Abstract

**Background:**

Previous studies have shown that acidic bleaching gels could lead to worse collateral effects during an in-office bleaching procedure, while neutral or basic products leads towards a better experience. Considering this fact, the main purpose of this study was to evaluate the pH behavior of 6 in-office bleaching gels, compared to the information provided by their manufacturers.

**Material and Methods:**

Thirty enamel discs of bovine teeth were prepared, the initial colors of which were measured by a spectrophotometer and then divided into 6 groups. A pH meter was used to measure the pH every 30 seconds until the end of each procedure, when a new color evaluation was then made. The Tukey test was used for statistical analysis of the results.

**Results:**

There was no difference in the color variation (ΔE) between the groups (*p*> 0.05). In two groups, the pH variation (ΔpH) showed neutral stability, with initial and final pH averages of 7.04 and 7.11 (*p* = 0.08) and 7.21 and 7.19 (*p* = 0.55), respectively; in another, there was alkaline stability, with an initial and final pH average of 8.54 and 8.37 (*p* = 0.14). In the other three brands, however, the results showed acidification, with initial and final pH averages of 6.14 and 5.22 (*p* = 0.001), 6.05 and 5.16 (*p* = 0.001) and 7.14 and 5.83 (*p* = 0.001), respectively.

**Conclusions:**

In 3 of the evaluated gels, a discrepancy existed between the manufacturer’s information and the data obtained, which could lead, considering previous studies discussed throughout this article, to unexpected collateral effects on the patients, especially dental sensitivity. Thus, clinicians and researchers should be aware about pH stability studies of in-office bleaching gels for better predictability and safety on their clinical usage.

** Key words:**Tooth bleaching, Bleaching agents, Hydrogen-ion concentration, Dentin sensitivity, Hydrogen peroxide.

## Introduction

The growing demand for cosmetic procedures intended to improve smile appearance, especially tooth whitening, has brought to the market a wide variety of whitening products for use by patients themselves, under the supervision of dentists, and by professionals in their office routine (1). The popularization of whitening techniques, whether used at home or in the office, owes to the safe and conservative nature of the procedure, as well as its simplicity and low cost, relative to other types of interventions (2).

Dental bleaching materials have hydrogen peroxide as the main active component, also found in the form of carbamide peroxide, which, during bleaching, dissociates into urea and hydrogen peroxide, accounting for about one-third of the initial concentration of the carbamide gel (3). During the bleaching process, the active component degrades into oxygen and water ions, and, through an oxidation reaction, will break the pigment molecules into smaller particles, thereby promoting tooth whitening (3).

Over the years, however, hydrogen peroxide has been shown to have the potential to promote certain adverse effects, not only in terms of tooth sensitivity (2,5), but also in several aspects of the morphological structure of the enamel, from increased porosity to decreased bond strength between restorations and previously conditioned enamel (5,6). More generally, however, even at high concentrations, hydrogen peroxide would not be able to cause morphological changes on the enamel surface (7-9), but the disparate pH levels of the bleaching gels would have the potential to promote such changes, especially in the interprismatic region, but, due to the presence of saliva, in a way that is reversible (10,11).

Brushing abrasion tests have shown that acidic gels have the potential to promote changes in enamel roughness and wear (10-12). In addition, evidence indicates that hydrogen peroxide gels with neutral pH tend to promote the same whitening result, when compared to more acidic gels, but have the advantage of reduced risk in the appearance and intensity of postoperative sensitivity (13).

Despite these data, there is still a great variability among products on the market, with pH levels ranging from the highly acidic to highly alkaline. However, many manufacturers do not bother to inform consumers about this property of their products (14). In addition, despite the launch of bleaching materials that describe themselves as “neutral” or “alkaline,” there is no evidence that demonstrates the ability of these gels to maintain a sTable pH throughout the bleaching procedure.

Thus, given the importance of the pH factor of whitening gels (10-13) and the amount of products already present and being launched on the market, the aim of this work was to evaluate the pH behavior of 6 different whitening gels used in in-office procedures, to study its variation in reaction with the dental structure, while evaluating the effectiveness of gels with different pH levels and verifying whether the evaluated initial values correspond to the values determined over the course of the procedure, while these gels are in contact with the dental enamel. The null hypothesis tested in this study is that there will be a tendency for all gels tested to acidify during their application to enamel.

## Material and Methods

-Specimen selection and preparation:

A total of 30 freshly extracted bovine incisors were selected for the study and stored in 0.05% Timol solution for up to 6 months to avoid collagen degradation. The teeth were cleaned by scraping the external surface with periodontal instruments and prophylaxis with pumice and water, after which the flatter areas of the buccal enamel portion of the bovine teeth were marked.

The standardization of the samples was carried out by planing the surfaces marked by sanding, in a metallographic polishing machine (model-APL-4 - Arotec, Cotia, Brazil), using abrasive silicon carbide sandpaper (600, 1200, 1500, 2000, 2500 and 3000) with abundant irrigation, avoiding dentin exposure during the procedure and aiming to maintain the enamel structure across the entire surface of all samples.

**Figure 1:**
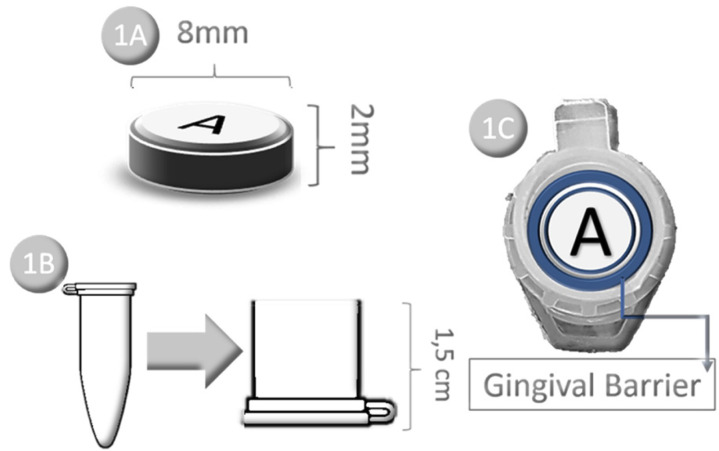
A) Illustration of bovine tooth disc after standardized cut; B) - Eppendorf cut 1.5 cm from the lid; C) Illustration of the gingival barrier for sealing the sample margins in the Eppendorf.

Then, discs of 8 mm diameter and 2 mm thickness were obtained (Fig. 1A), by clipping the fragments with cylindrical diamond tips and a high-speed turbine, adapted from standard equipment for cavity preparations.

After obtaining the samples, the initial color registration of each fragment was performed using a spectrophotometer (Model CM 2600D - Konica Minolta, Tokyo, Japan). Then, Eppendorf tubes were cut at a height of 1.5 cm from the lid, to provide an opening through which the whitening gel could be safely applied without overflow (Fig. 1B).

With the tubes cut and supported on a flat surface, the tooth fragments were positioned on the tube caps, with the enamel portion facing upwards. After positioning, light-curing resinous fluid (TopDam - FGM, Joinville, Brazil) was applied to the margins of the samples, to prevent the whitening gel from infiltrating into the dentin portion, mimicking the clinical practice, according to which, the whitening gel only come into contact with dental enamel during bleaching (Fig. 1C).

The samples were then subjected to the bleaching procedures in the subsequent steps.

-Dental bleaching and pH assessment:

The prepared specimens were randomly divided into 6 groups (n = 5), according to the bleaching gel to be used. All gels were applied pursuant to the instructions indicated by their respective manufacturers (Table 1).

**Table 1:**
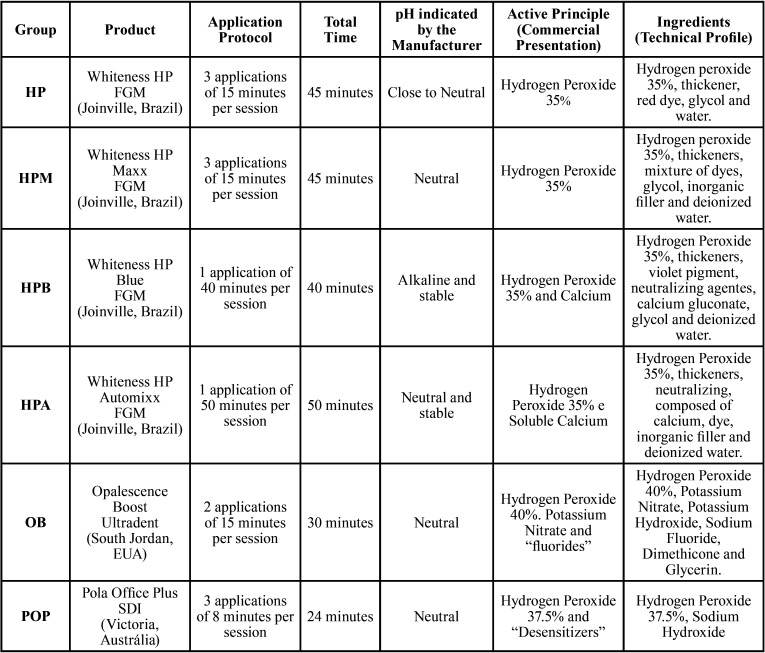
Evaluated groups characteristics: Composition, usage instructions and pH characteristics indicated by the manufacturers.

To evaluate the pH levels of the whitening gels, a portable pH meter with a digital indicator (Model 3611 - Spencer) and a rechargeable pH electrode (Model V621 / 175mm - Analion) of small dimensions, compatible with the sample size, were used. Before each session, the equipment was calibrated according to the manufacturer’s recommendation, first with an acidic pH substance (pH 4.0) and then with a neutral pH substance (pH 7.0).

**Figure 2:**
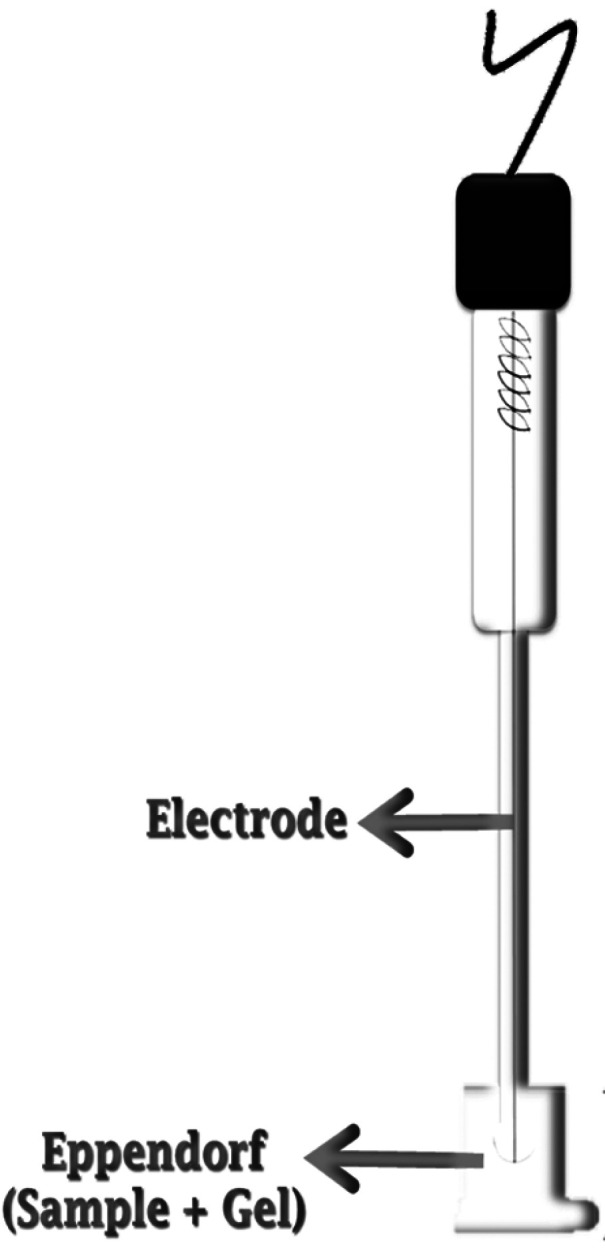
Immersion of the electrode in the gel dispensed in the Eppendorf structure with the bovine tooth sample.

Due to limitations of the instrument itself, it was necessary to apply a layer of gel 5mm thick, to enable complete immersion of the electrode tip in the solution, allowing for correct pH measurement with minimal waste. After the tip was immersed in the gel, the values were recorded every 30 seconds, over the entire application time, as indicated by the manufacturer of the gel used, totaling the time corresponding to an in-office whitening session (Fig. 2).

After each measurement, the electrode was washed generously with distilled water, and then dried with absorbent paper, as recommended by the device manufacturer. This sequence was repeated with each immersion of the electrode tip in the gel, to avoid possible changes in the results. When the gel was replaced, per the manufacturer’s instructions, during the bleaching procedure, it was done following the respective time instructions and the cleaning of the samples. Subsequently, the application of peroxide followed the protocol recommended by the manufacturers of each gel. 

-Data analysis:

After the pH measurement step, the samples were evaluated again, by spectrophotometer, to record their final colors. All data were tabulated and evaluated statistically. ANOVA statistical analysis of repeated bidirectional measurements and a subsequent Tukey test, were conducted, yielding a significance level of 5%, for color analysis (ΔE) as well as for pH analysis (ΔpH), using the software SPSS version 21.0 for the Microsoft Windows operating system (IBM Analytics - USA).

## Results

The color variation averages promoted by each gel can be seen in Table 2. Regarding the color variation of the samples (ΔE), no statistical difference was detected between the groups evaluated (*p*> 0.05), regardless of the pH found.

**Table 2:**
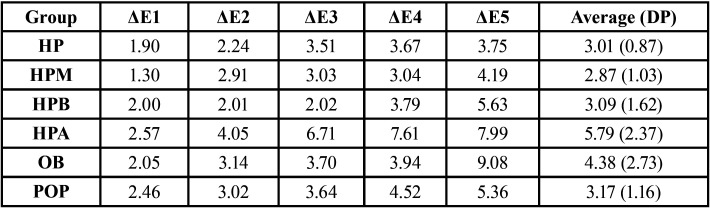
Average color variation and standard deviation.

As for the pH variation, the results showed neutral stability in the gels Opalescence Boost (Ultradent) and Pola Office Plus (SDI), alkaline stability in the Whiteness HP Blue gel (FGM), and tendency toward acidification in the other 3 gels evaluated (Table 3).

**Table 3:**
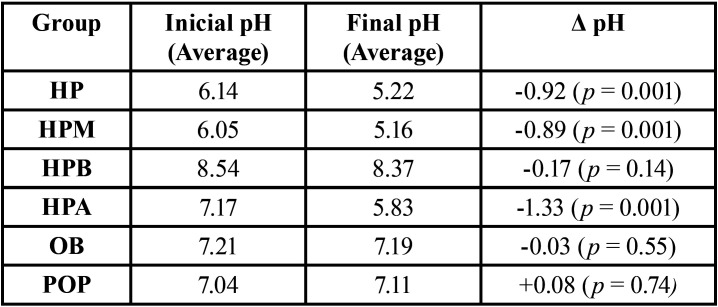
Average of Initial, Final and Δ pH.

**Figure 3:**
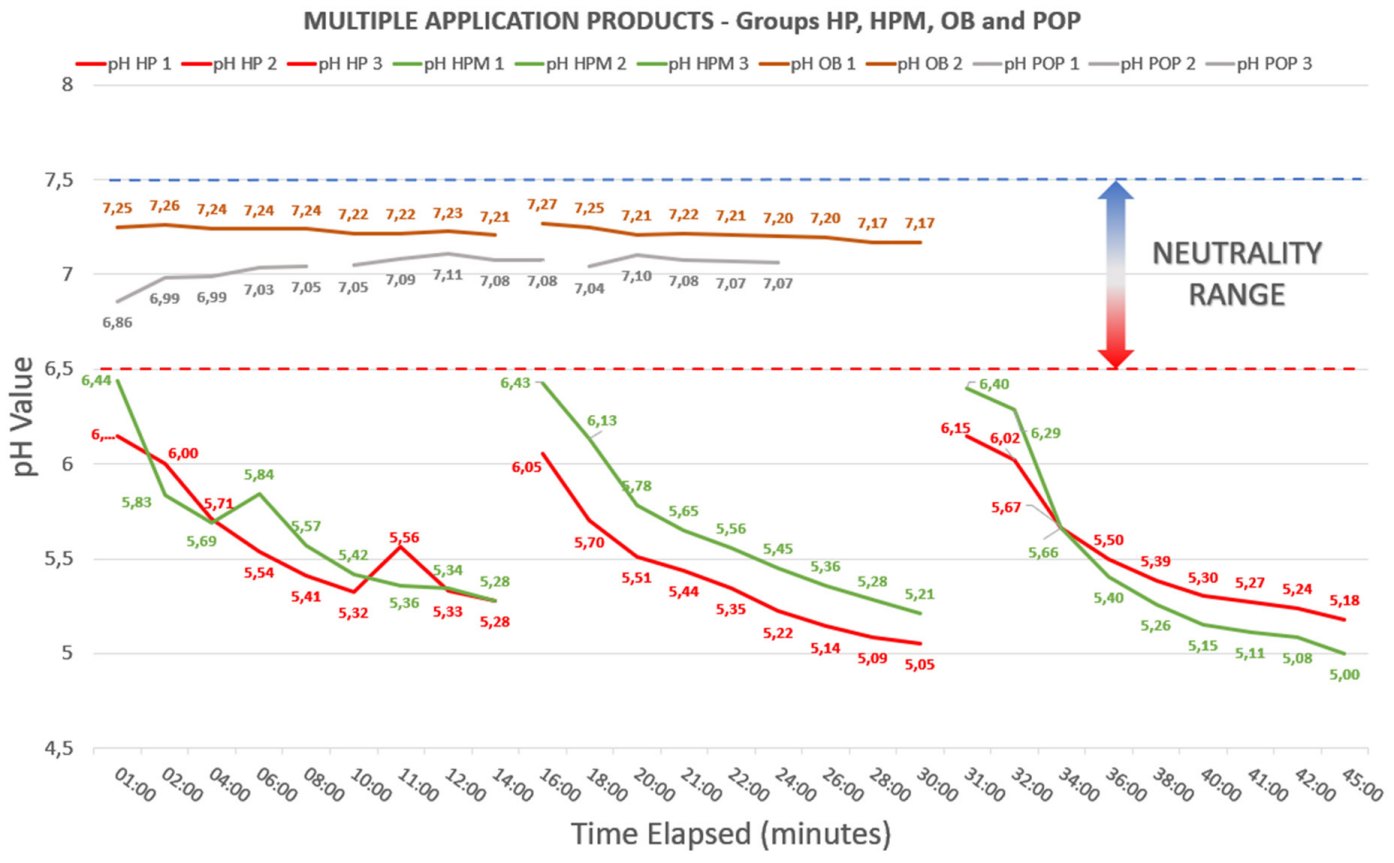
Multiple Application Products and their pH variation curves. The results shown are from Whiteness HP (FGM), Whiteness HP Maxx (FGM), Opalescence Boost (Ultradent) and Pola Office Plus (SDI) – Groups HP, HPM, OB and POP, respectively.

**Figure 4:**
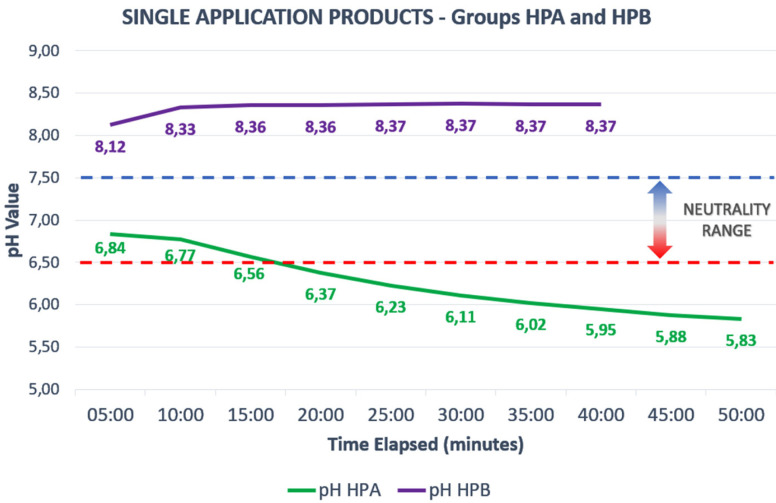
Single Application Products and their pH variation curves. The results shown are from Whiteness HP Automixx (FGM) and Whiteness HP Blue (FGM) – Groups HPA and HPB respectively.

The pH variation curve of each gel, over the entire period that it was in contact with the enamel, can be seen in Figures 3,4, where the average pH is noted at each measurement time for each gel, in each of the 5 specimens tested for each group.

## Discussion

Currently, there is a great deal of discussion about the action and effect of whitening gels on the tooth structure, depending on their specific chemical properties (5-15), but all results, even when positive in the laboratory, have been shown to be irrelevant during clinical practice. Such evidence may indicate that, regardless of the pH of the bleaching gel, there will be no irreversible damage to the enamel structure during dental bleaching (11).

A salient concern, however, regards the tooth sensitivity commonly reported after this type of treatment (2,4,11). The use of previous medication, such as dexamethasone (16), ibuprofen (17), dipyrone for topical use (18), among other drugs, has not been shown to be effective in preventing dental sensitivity after office bleaching. Although several experimental products have been tested, with favorable results (19), the substance that has, to date, been most effectively evaluated, in terms of sensitivity control, is 5% potassium nitrate in gel form, used after tooth whitening, and even this treatment merely eases these effects (20-22).

As a result, many studies are still being conducted to define ways to prevent or mitigate this type of side effect before it can even occur, such as through the use of different concentrations of whitening gel (23,24). This aspect of the substance, however, is difficult to modify when it comes to in-office whitening, given the circumstances in which it is indicated and the need for a higher hydrogen peroxide concentration in its composition to achieve the expected results. Thus, it is important to search for alternative and additional means to promote to the prevention or reduction of these adverse effects.

In view of this problem, the results of this study may be said to call attention to the pH issue, not only through its evaluation of the information provided by the manufacturers of the bleaching gels, but mainly for its recognition of the importance of neutrality in controlling the side effects. For example, pH can be used as an additional way to control tooth sensitivity during and after the procedure. Clinical studies have already shown that there is, in fact, less of a tendency toward and less intensity of sensitivity when using neutral or alkaline pH gels, compared to acid pH gels (4,12,25,26).

There is evidence that this may be due to the increased formation of ions due to the higher pH, which generates more free radicals (27) that would be associated with a faster decomposition of hydrogen peroxide (28) and, consequently, according to the theory that currently enjoys the widest acceptance regarding the etiology of sensitivity caused by tooth whitening, lower chances that the agents will have direct contact with the neuronal tooth pulp receptors during the reaction of the whitening gels (13,19). This possibility has actually been reinforced by recent research, which showed a greater penetration power of acidic hydrogen peroxide gels and their concentration inside the pulp chamber after tooth whitening, relative to gels with a more neutral or basic pH (29).

Regarding the behavior of gels, despite the literature pointing to a general tendency for bleaching gels to become acidic during their application to bovine teeth, (12) the analyses in this study revealed that there are gels with the ability to maintain a stable pH throughout the time of its application, which meant that our null hypothesis was rejected, because not all gels tested showed the expected acidification tendency. A worrying factor, however, was the finding that, in at least 3 of the 6 gels evaluated, the manufacturer’s information on the neutrality and stability of the gels was mistaken, which can induce professionals to pass along mistaken information on the products to their patients. An important fact to mention, regarding the observed pH behavior of these tested gels, is that they tended to become acidified from the beginning of their application (Fig. 3). When comparing these results with results found in previous studies, (12,30) it appears that the values found in the measurement of the Whiteness HP Maxx and Whiteness HP Blue gel, as well as of the Opalescence Boost, were similar to those found in our measurements, which further proves the reliability of the methodology used and, consequently, of the results obtained. 

Other studies (31) showed similar behaviors of the pH from Whiteness HP Maxx and Whiteness HP Blue, with additional information about the decomposition rate of the hydrogen peroxide concentration, on which stable neutral gels are indicated as less prompted to have their peroxide concentration decreased throughout time, being able to be clinically applied for more time with the same efficacy when compared to their removal from time to time technique as specified by their manufacturers. This could be applied, for example, on Opalescence Boost and Pola Office Plus, as our results showed a tendency for pH stability during their application. Therefore, there is evidence, due to their neutral and stable behavior, that these gels don’t need to be exchanged during the in-office procedure, which means that manufacturers should review their application protocols for clinical usage, indicating higher application times without so changes during a bleaching session.

On the other hand, Whiteness HP Automixx is indicated for 50 minutes straight application, which leads us to believe on their neutral stability as said by its manufacturer. However, on the present study, its pH behavior showed to be unstable compared to the other gels, including its predecessor, Whiteness HP Blue, which pH behavior was completely stable through all the application time. That indicates that, differently from Pola Office Plus, Opalescence Boost and Whiteness HP Blue, the Whiteness HP Automixx manufacturer should change its protocol of application to the exchanging method through time, in order to keep its pH in the neutrality range and, therefore, its bleaching efficacy with less risk of tooth sensitivity.

An important fact about the previous studies mentioned on this article (12,30), is that they performed pH measurements differently from one another. One study (12) conducted the measurements only at the beginning and the end of the application of the bleaching gels, according to the manufacturers’ recommendation, leaving them unable to generate a pH variation curve for analysis, while the other (30) measured the pH every 2 minutes for 45 minutes, regardless of the manufacturer’s recommendation, which could be considered as a bias in our study, given our objectives. In addition, it is important to mention that only in the first of these studies (12) did the gels make contact with bovine tooth enamel during analysis, a factor that was considered extremely important, due to the ionic exchanges that occur as while peroxide is acting on the dental structure, which may, in theory, cause differences in pH variation. Another important factor is based on the fact that the presence of the dental structure is essential to prove the activity of the whitening gel, and yields necessary data for the establishment of true conclusions regarding the behavior and characteristics of the evaluated products.

To adapt the methodology to the objectives of this study, obtaining a pH variation curve and thus establishing the level of stability of the evaluated office bleaching gels, it was determined necessary evaluate the pH change every 30 seconds , for the full application period of the gel indicated by its manufacturers. Through this method, it was possible to evaluate the data more effectively, making it possible to determine which products change their pH during use and while in contact with tooth enamel; it also allowing for observation of the point at which the pH of gels started to change too much to be considered as “neutral,” and would more appropriately be classified as acidic or basic, depending on the values obtained. For this purpose, it was established that pH values between 6.5 and 7.5 fell within the neutrality range, with 7 being the absolute neutral value.

In terms of the color variation between the groups, it was assessed that there was no statistically significant difference in the color variation of the samples (ΔE), which is consistent with the results found in other studies, which have already stated that the ability of the gels to promote whitening does not depend on its pH (13,25,26). In addition, such results support the idea that it is unnecessary use more acidic products to obtain better immediate teeth whitening results. In this study, color evaluation was necessary to verify the activity of the bleaching gels while they were in contact with the dental structure, to positively demonstrate that ion exchange occurred through oxidation of the pigmentation particles in the specimens used, thus supporting our objectives.

In one study (25), the initial pH of the brands Whiteness HP Maxx and Whiteness HP Blue gels (FGM, Brazil) were relatively higher, relative to the results found in this study, while the initial pH of Opalescence Boost (Ultradent, USA) was slightly lower. This may be attributed to the presence of tooth structure in this study during the evaluation of bleaching gels. Despite this, the behavior of these gels over time has been shown to be similar in both studies. The presence of dental structure during the analysis represents a better laboratory mimicry of clinical practice, a relevant factor for the control of possible biases in this type of study. However, the influence of these ionic exchanges on the pH variation of bleaching gels during tooth whitening still needs further investigation to produce a better correlation.

Given the facts presented, therefore, and based on the results of the present study, to promote the main benefit of neutral pH, in terms of the reduction of side effects, especially post-bleaching sensitivity, it is important that the manufacturers of bleaching gels conduct measurements of the pH variation of their products, creating suitable protocols to maintain the pH within the neutral range, promoting the periodic exchange of the mixture in contact with the dental structure, a practice that is already carried out by some commercial brands. Such conduct will allow clinicians and their patients to enjoy the benefits inherent in neutral pH gels and, in theory, those of basic pH, in terms of preventing absolute risk and intensity of tooth sensitivity after office bleaching.

In addition, this study demonstrates the importance of getting correct information from manufacturers, regarding the pH behavior of their whitening gels, to ensure that dentists understand and better apply the protocols established for each product. Therefore, clinicians and researchers should be attentive to studies regarding the stability and pH measurement of in-office bleaching gels to avoid possible errors of clinical indication and optimization in the sensitivity control of their patients in procedures performed with high-concentration hydrogen peroxide gels. More clinical studies about the pH of bleaching gels, associated with tooth sensitivity, should be done, however, given the measurement in previous studies of the pH of bleaching gels already evaluated for use, to guarantee the correct analysis of the data obtained in the research.

Based on the results of the present study, and given the limitations intrinsic to an *in vitro* study, it was initially concluded that there were no significant changes in the efficacy of whitening, regardless of the pH of the gel used. In addition, it was observed that, in 3 of the 6 gels evaluated, the pH information furnished by the manufacturers did not correspond to that determined by the *in vitro* measurements, and also noted that the gels with a tendency toward acidification begin the process of dropping in pH from the beginning of its application, thus reinforcing the importance of control, correct indications, and application of office bleaching gels by manufacturers, as well as by and dentists in their clinical practice.
